# Stigma and level of familiarity with opioid maintenance treatment (OMT) among specialist physicians in Israel

**DOI:** 10.1186/s12954-023-00869-9

**Published:** 2023-09-15

**Authors:** Rozner Lihi, Delayahu Yael, Brill Silviu, Sason Anat, Weinstein Marsha, Shoshan Stacy, Schreiber Shaul, Adelson Miriam, Peles Einat

**Affiliations:** 1 Department of Psychiatry, Maayenei Hayeshua Medical Center, Bnei Brak, Israel; 2 Psychiatry, Abarbanel Mental Health Center, Bat Yam, Israel; 3 Faculty of Medicine, Tel Aviv University, Tel Aviv, Israel; 4 Pain Institute, Tel Aviv Sourasky Medical Center, Tel Aviv, Israel; 5 Adelson Clinic for Drug Abuse Treatment and Research, Tel Aviv Sourasky Medical Center, 10 Dafna Street, 6492805 Tel Aviv, Israel; 6 Division of Psychiatry, Tel Aviv Sourasky Medical Center, Tel Aviv, Israel

**Keywords:** Opioids, Stigma, Approach, Knowledge, Opioid maintenance treatment, Opioid use disorder

## Abstract

**Context:**

Opioid use disorder (OUD) poses significant public health problems that have increased dramatically, resulting in high rates of morbidity and mortality.

**Objectives:**

To minimize the risk of an opioid epidemic in Israel and be prepared, we evaluated physicians’ objective knowledge, level of stigma, and approach to prescribing opioids, risk factors, and identification of patients with substance use disorder (SUD), as well as their knowledge about opioid maintenance treatment (OMT) for OUD.

**Methods:**

Anonymous computerized questionnaires were distributed nationally to physicians by the Israel Medical Association. Knowledge, stigma, and approach were scored.

**Results:**

Of only 249 responders, 58.6% prescribe opioids, 32.1% prescribe cannabis, and 18.5% daily encounter patients with SUD. Logistic regression found the high knowledge group had daily encounters with SUD (Odds Ratio (OR) = 3.5, 95% CI 1.7–7.1) and were familiar with OMT (OR = 10.1, 95% CI 3.5–29.0). The high stigma group was characterized by physicians who prescribe opioids (OR = 1.7, 95% CI 1.0–2.9), but who self-reported having limited knowledge regarding OMT (OR = 2, 95% CI 1.1–3.7). The high approach group was characterized by those who prescribe opioids (OR = 11.7, 95% CI 4.9–28), prescribe cannabis (OR = 2.1, 95% CI 1.0–4.3), self-report having limited knowledge regarding OMT (OR = 11.2, 95% CI 1.4–89) and self-report identifying SUD (OR = 32.5, 95% CI 4.1–260).

**Conclusion:**

High stigma was most evident among physicians who prescribe opioids but, importantly, who had limited knowledge specifically regarding OMT. Gaps in knowledge and approach were observed. An educational intervention is highly recommended to reduce stigma and increase referrals of patients for OMT, the most effective treatment for opioid use disorder**.**

**Supplementary Information:**

The online version contains supplementary material available at 10.1186/s12954-023-00869-9.

## Introduction

Opioids are often prescribed as part of acute and chronic pain management [1]. However, opioid use disorder (OUD) (see below) has posed significant socioeconomic and public health problems that have increased dramatically over the past three decades, resulting in high rates of morbidity and mortality [2–5]. The US government has declared the opioid epidemic a national emergency [3, 5]. Pursuant to this, the US Congress has presented strategies for addressing the problem, including an educational campaign, easier access to non-opioid treatments and medications for chronic pain, and the establishment of an electronic prescription drug reporting program [3, 5]. Out of all Organization for Economic Co-operation and Development (OECD) nations, only Israel saw opioid prescribing double between 2011 and 2013, and up until 2014–2016 [6]. Moreover, a study that evaluated opioid prescription in Israel’s largest healthcare payer/provider organization reported an increase in opioid prescribing to adult non-cancer pain patients under age 65 between 2008 and 2018 [7]. At the same time, the national mortality rate did not increase between 2005 and 2014 [8], and up until 2018 [9]. In 2020, for the first time, Israel surpassed the USA and became the country with the highest consumption of prescription opioids in the world [10].

Opioid use disorder (OUD) is a chronic relapsing brain condition, whose management requires continuous treatment for those patients who suffer from it [11]. Opioids may serve as an effective treatment for acute pain, but are a questionable response to chronic pain [12–15]. Reducing opioid prescriptions is a big challenge in pain medicine [3]. Opioid use disorder is a chronic disease characterized by the persistent use of opioids despite harmful consequences. Patients typically have both physical dependence and loss of control over their opioid use, and may experience serious medical, social, financial and familial consequences related to their use. It is a relapsing disorder, with increased risk of reverting to opioid use even after years of abstinence [16]. Most people who have developed OUD will need long-term OMT with either methadone or buprenorphine [12, 15]. The Israel Ministry of Health subsidizes SUD treatment services and rehabilitation, including public OMT programs. It has been hypothesized that stigmatization that can lead to fear, shame, and social isolation [17–19] may hamper or delay referrals to OMT programs (i.e., ignorance and stigma against OMT among social services department personnel [20]). A delay occurs even though it is the most effective treatment option for the majority of individuals with OUD [20–23].

In addition, since 2013, cannabis has been legally prescribed in Israel for 12 specific medical indications in various fields, among them psychiatry, pain management, and palliative care. Physicians who are trained and certified by the Israel Ministry of Health may prescribe cannabis to patients with one or more of these indications [24], and thus physicians’ knowledge, attitude and approach regarding cannabis is of interest, and may be relevant to their knowledge and attitude regarding opioids.

Several recent studies have reported on stigma [25], or stigma and approach [26]. A review of 37 studies between 2015 and 2021 examined how physicians’ perceptions or stigma influenced harm reduction efforts and addressed clinical knowledge gaps in overdose treatment and prevention and OUD treatment [27]. They reported that less than half of the studies addressed access issues at the system level, above the individual healthcare professional. We evaluated self-reported perceived knowledge, and administered an objective knowledge questionnaire that examines in depth the gaps in knowledge that may lead to stigma.

We hypothesized that gaps in prescription and follow-up by physician specialization may be at the root of the increasing number of patients developing OUD. Specifically, a surgeon may prescribe opioids for acute pain, but then may not follow-up with the patient, and refer them to the family physician or internal medicine specialist in an outpatient setting, who will not necessarily change the dosage or stop their medication. At the same time, pain physicians may prescribe opioids for chronic pain—while the consequences of OUD may then be addressed by a psychiatrist. Further complicating the situation is the fact that only those physicians who specialize in addiction may refer OUD sufferers to OMT. Given the absence of any published report and our desire to prepare to address the looming threat of an opioid use epidemic in Israel, we conducted a national survey of all physicians in Israel. To this end, we created a new tool to evaluate this domain. Our aims were to evaluate physicians’ knowledge, stigma, and approaches with respect to opioids—specifically, their familiarity with SUD risk factors, their ability to identify SUD in patients, and their ability to differentiate between physical dependence and substance use disorder. Our study also aimed to evaluate physicians’ opioid prescribing habits (indication, duration, dosage, and frequency); management of patients who have been prescribed opioids (evaluation of risk factors for substance use disorder, and limitation and monitoring of usage); stigma toward substance use disorder; and holding of stigma-erroneous information and beliefs toward OMT with methadone or buprenorphine. We were also interested in verifying whether physicians’ self-reported perceived knowledge was confirmed by an objective evaluation of their knowledge.

## Methods

This national survey among all physicians who practice medicine in Israel was approved by the IRB of the Tel Aviv Sourasky Medical Center.

1. Study population: All 18,651 physicians registered with the Israel Medical Association (IMA) were eligible for inclusion in the survey, with no exclusions. A computerized questionnaire was transmitted via the IMA e-mail distribution system to all physicians in the country, who were asked to anonymously complete it via an internet link. Concurrently, inquiries were made to healthcare organization leaders, and intensive attempts were made to distribute the questionnaire among physicians. In addition, interviews were conducted using the same questionnaire among 50 physicians, most of them in managerial positions from relevant specialties (addiction, pain medicine, etc.) who work at various hospitals and in the community. We personally e-mailed them one by one, asking their consent to contact and arrange for an in-person structured interview using the same questionnaire. They all self-reported not having participated in the anonymous survey.
2. Research tools: An initial questionnaire was constructed in collaboration with experts in the fields of pain medicine, addiction medicine, and psychiatry. To achieve face validity, the questionnaire was revised after receiving feedback from physicians working in other fields (surgery, gastroenterology, and internal medicine), and the final version was formulated (see questionnaire-Additional file 1: appendix). The questionnaire included questions regarding knowledge, stigma, and approach (dependent variables), along with an overall score for all three components. The knowledge section included 32 questions about diagnosing, risk factors for developing substance use disorder, and treatment and referral options for patients who suffer from substance use disorder. The section on stigma used eight questions to examine physicians’ beliefs regarding the treatment of patients who suffer from SUD. The approach section included questions about the rate of prescription of opioid medications, whether the risk of developing a SUD was considered, whether follow-up was conducted for patients who were prescribed opioids, whether SUD was identified and, if so, whether those individuals were referred for treatment at the appropriate facilities. Respondents were asked to answer on the Likert scale between strongly disagree (1) and strongly agree (5) (Cronbach α was 0.89 for knowledge and 0.65 for stigma). For a correct answer on questions concerning knowledge, both 4 and 5 (agree or strongly agree) were accepted. The approach score included 13 questions (see questionnaire-Additional file 1: appendix), which were included in the score if the physicians responded to questions. The percentage of correct answers out of all the answers was calculated for a total score on a scale of 0–100. Internal consistency (reliability) for three subgroup questions was estimated using half split to achieve a Spearman Brown coefficient of 0.75, 0.76 and 0.71 for knowledge, approach and stigma, respectively.
3. Independent variables included physicians’ sociodemographic background (age, gender, country of birth, year of immigration to Israel); professional characteristics (the country in which they studied medicine, medical specialization, treatment framework (community/ hospital), seniority, position, scope of work); the level of exposure to individuals with SUD (of opioids and/or cannabis); prescription of opioids (yes or no) and/or cannabis (yes or no); and familiarity (rated between 1 and 5) with 1. Opioid treatment for chronic non-cancer pain, 2. Identification and diagnosis of addiction disorder, 3. Opioid addiction treatment options, 4. Methadone maintenance, and 5. Buprenorphine maintenance.

### Statistical methods

The IBM SPSS Version 25.0 was used. Chi-square or Fisher exact tests were used for categorical variables, and the t test or ANOVA were used for continuous variables (means ± standard deviations are presented). The dependent variables (knowledge score, stigma, and approach) were compared by independent variables, some of which were collapsed into two or three categories (yes or no for “encounter SUD patients daily,” “prescribe opioids,” “prescribe cannabis,” and “familiarity with several subjects”). A comparison between high scores (75th percentile) to all other scores (“low”) for each of the three dependent variables was performed. Multivariate analyses were done using ANCOVA for continuous variables (global scores). Logistical regression models were used for low and high categorical scores, including the independent variables that were significant (*p* < 0.1) in univariate analyses.

## Results

### Characteristics of the study group

18,651 emails were sent to all Israel Medical Association physician members. 7291 of them opened the e-mail, 274 clicked to enter the survey site, and only 199 filled out the anonymous questionnaires. Together with 50 personal interviews, a total of 249 physicians answered the questionnaire (1.3% of 18,651 emails sent, 3.4% of the 7291 who opened the e-mail). The characteristics of the study group are presented in Tables 1, 2. Of them, 45% were women, 81.9% were specialists, 58.6% prescribed opioids (ranging from 1 to ≥ 20 prescription per month), and 32.9% prescribed cannabis for medical use. Daily encounters with patients who suffer from SUD were reported by 18.5% of the respondents. Of all specialties, psychiatry was most prevalent (24.9%) (Table 2) and represented the highest proportion of physicians who encountered SUD patients daily (37.1%). All the oncologists and almost all the family physicians reported prescribing opioids, while 90.9% of pain medicine physicians reported prescribing cannabis.

**Table 1:** Participants’ characteristics

*N* (%)	249 (100)
Anonymous	199 (79.9)
Female	112 (45.0)
Age (years) mean ± S.D	50.6 ± 12.7
Seniority (years) mean ± S.D	20.0 ± 13.4
Manager	127 (51.0)
Full time job	207 (83.1)
Studied in Israel	182 (73.1)
Specialist	204 (81.9)
*Working place*	
Community	70 (28.1)
Hospital	110 (44.2)
Both	69 (27.7)
*Opioid prescription per month*	
No	103 (41.4)
1-5	67 (26.9)
6-20	50 (20.1)
≥ 20	29 (11.6)
*Opioid indication (10 not reported)*	
Cancer	49(33.6)
Acute pain	53(36.3)
Chronic pain	34(23.3)
*Cannabis prescription per month *	
No	167 (67.1)
1-5	52 (20.9)
6-20	21 (8.4)
≥ 20	9 (3.6)
*Opioid and Cannabis prescription*	
No	75 (30.1)
Opioid	92 (36.9)
Cannabis	27 (10.8)
Both	55 (22.1)
*Encounters with SUD*	
Daily	46 (18.5)
Several per month	88 (35.3)
Several per year	83 (33.3)
None	32 (12.8)

**Table 2:** Specialty distribution and proportion of prescribe opioid, cannabis, and daily encounter SUD by each specialty

*N*	249 (100%)	Prescribe opioids	Prescribe cannabis	Daily encounter SUD
*Specialty*				
Psychiatry	62 (24.9)	9 (14.5)	18 (29.0)	23 (37.1)
Surgery	31 (12.4)	24 (77.4)	4 (12.9)	4 (12.9)
Internal medicine	48 (19.3)	34 (70.8)	9 (18.8)	11 (22.9)
Pain medicine	22 (8.8)	18 (81.8)	20 (90.9)	4 (18.2)
Family medicine	34 (13.7)	33 (97)	11 (32.4)	2 (5.9)
Oncology	6 (2.4)	6 (100)	5 (83.3)	0
Geriatric/rehab/anesthesia	25 (10)	16 (64.0)	8 (32.0)	1 (4.0)
Neurology	10 (4.0)	3 (37.5)	6 (60.0)	0
Other	3 (1.2)	0	0	0
Pediatrics	8 (3.2)	0	1 (12.5)	1 (12.5)

### ***Self-reported knowledge (***Table 3***)***

**Table 3:** Self-reported level of familiarity with specific subjects

Subject	N	Treat using opioids for non-cancer chronic pain	Identify substance use disorder	Treatment options for opioid use disorder	Methadone maintenance treatment (MMT)	Buprenorphine maintenance treatment
Total	249	**2.9 ± 1.4**	**3.1 ± 1.3**	**2.7 ± 1.2**	**2.3 ± 1.2**	**2.0 ± 1.2**
*p value (*F***)**		**< 0.001** (15.1)	**< 0.001** (6.7)	**< 0.001** (5.2)	**< 0.001** (4.2)	**< 0.001 **(4.6)
*Specialty*						
Psychiatry	62	2.1 ± 1.3	3.6 ± 1.2	3.1 ± 1.3	2.6 ± 1.2	*2.6* ± *1.3*
Surgery	31	2.2 ± 1	2.3 ± 1.1	1.8 ± 0.8	1.4 ± 0.6	1.3 ± 0.5
Internal medicine	48	3.1 ± 1	3.0 ± 1.1	2.5 ± 1.0	2.6 ± 1.2	1.8 ± 1.1
Pain medicine	22	*4.7* ± *0.5*	*4.0* ± *1.0*	*3.5* ± *1.1*	*2.8* ± *1.3*	*2.6* ± *1.3*
Family medicine	34	3.7 ± 1.1	3.5 ± 1.1	2.9 ± 1.2	2.2 ± 1.1	1.9 ± 1.2
Oncology	6	2.5 ± 1.6	2.7 ± 1.6	3.0 ± 1.3	2.2 ± 1.5	2.0 ± 1.5
Geriatric/rehabilitation/anesthesia	25	2.9 ± 1.3	2.7 ± 1.3	2.2 ± 1.1	2.0 ± 1.2	1.6 ± 0.9
Neurology	10	3.6 ± 1.1	2.5 ± 1.2	3.2 ± 1.0	1.7 ± 0.9	1.8 ± 1
Other	3	2.7 ± 2.1	2.3 ± 2.3	2.3 ± 2.3	2.3 ± 2.3	2.3 ± 2.3
Pediatrics	8	2.1 ± 1.5	2.4 ± 1.6	2.3 ± 1.6	1.8 ± 1.5	1.4 ± 1.1

^*^ANOVA, multiple comparison corrected model *p*(*F*)

Mean scores on the degree of self-reported knowledge about opioid- and substance use disorder-related issues (on a scale of 1 “no knowledge at all” to 5 “excellent knowledge”) were low. Scores ranged from a mean of 3 for the ability to detect pain and substance use disorder (a score of 6 on a scale of 1–10), to a mean score of 2 concerning OMT with methadone or buprenorphine (a score of 4 on a scale of 1–10). Overwhelmingly, pain medicine physicians reported having wide knowledge of all subjects (Table 3). Self-reported ability to identify SUD was greatest among pain physicians, psychiatrists, and family medicine physicians, who reported having a high knowledge of these topics, with the rest of the respondents scoring below the mean (3.1 ± 1.3**)**.

### Objective knowledge

#### *Knowledge test score (*Table 4*)*

**Table 4:** Knowledge, stigma, and approach scores, by selected variables

	*N* (%)	Knowledge score	*p* value	Stigma score	*p* value	Approach score	*p* value
All sample	249 (100%)	48.5 ± 17.9		25.7 ± 19.6		31.7 ± 22.8	
*Medicine studied*			0.06		0.5		0.5
Israel	182 (73.1)	49.8 ± 18.1		26.2 ± 19.5		31.1 ± 21.6	
Others	67 (26.9)	45.0 ± 16.8		24.4 ± 20.0		33.3 ± 25.8	
*Gender*			0.1		0.8		0.6
Female	112 (45.0)	50.3 ± 16.7		25.3 ± 20.0		30.8 ± 22.1	
Male	137 (55.0)	47.0 ± 18.7		26.0 ± 19.4		32.5 ± 23.4	
*Age group*			0.2		0.8		0.05
< 45	86 (34.5)	49.1 ± 16.3		25.1 ± 20.1		30.5 ± 22.0	
45–64	122 (49.0)	49.6 ± 18.8		25.5 ± 20		34.8 ± 23.3	
65 +	41 (16.5)	44.1 ± 17.9		27.4 ± 17.7		25.1 ± 21.5	
*Manager*			0.2		0.2		0.05
Yes	127 (51)	49.8 ± 17.5		24.2 ± 19.9		34.5 ± 23.2	
No	122 (49)	47.2 ± 18.3		27.3 ± 19.3		28.9 ± 22.0	
Encounter daily			**0.0005**		0.5		0.07
*SUD patients*							
Yes	46 (18.5)	59.3 ± 15.9		27.4 ± 22.7		37.3 ± 18.6	
No	203 (81.5)	46.1 ± 17.4		25.3 ± 19.2		30.5 ± 23.5	
*Prescribe opioids*			0.7		**0.03**		**< 0.0005**
Yes	146 (58.6)	48.8 ± 16.6		27.9 ± 20.8		39.8 ± 23.4	
No	103 (41.4)	48.1 ± 19.7		22.6 ± 17.5		20.3 ± 16.1	
*Prescribe cannabis*			**0.002**		0.9		**< 0.0005**
Yes	82 (32.9)	53.5 ± 16		25.8 ± 20.5		39.5 ± 20.9	
No	167 (67.1)	46.1 ± 18.3		25.7 ± 19.2		27.79 ± 22.7	
*Specialty*			**0.0005**		0.1		**< 0.0005**
Psychiatry	62	56.8 ± 16.8		20.8 ± 14.1		23.3 ± 15.5	
Surgery	31	39.9 ± 18.6		25.4 ± 24.5		19.6 ± 20.8	
Internal medicine	48	46.9 ± 14.6		28.6 ± 20.3		34.9 ± 22.0	
Pain medicine	22	57.2 ± 14		34.1 ± 22.6		50.7 ± 20.8	
Family medicine	34	51.5 ± 16.3		29.8 ± 19.2		49.8 ± 21.8	
Oncology	6	41.1 ± 16.0		14.6 ± 12.3		28.2 ± 16.6	
Geriatric/rehab/*	25	39.4 ± 17.7		23.5 ± 20.5		27.7 ± 22.0	
Neurology	10	46.3 ± 16		20.0 ± 13.4		18.5 ± 14.6	
Other	3	40.6 ± 14.3		37.5 ± 33.1		35.9 ± 24.7	
Pediatrics	8	31.3 ± 22.3		25.0 ± 20.0		26.0 ± 29.6	
*Working place*			0.5		0.9		0.1
Community clinic	70 (28.1)	49.2 ± 18.0		26.7 ± 18.2		35.2 ± 24.7	
Hospital	110 (44.2)	47.0 ± 16.9		26.1 ± 21.3		28.5 ± 20.8	
Both	69 (27.7)	50.3 ± 19.3		24.6 ± 18.6		33.3 ± 23.4	
*Self-report: identify*			**0.0005**		0.05		**< 0.0005**
No	39 (15.7)	34.7 ± 14.2		19.9 ± 17.6		12.7 ± 12.7	
Partial	106 (42.6)	47.7 ± 15.7		25.0 ± 18.5		30.0 ± 22.2	
Yes	104 (41.8)	56.0 ± 15.4		28.6 ± 21.0		40.8 ± 21.3	
*Self-report: OMT*			**0.0005**		**0.009**		**< 0.0005**
No	83 (33.3)	39.7 ± 15.6		22.7 ± 18.6		21.9 ± 19.9	
Partial	124 (49.8)	52.4 ± 14.9		29.4 ± 20.3		34.9 ± 23.1	
Yes	42 (16.9)	58.3 ± 16.8		20.5 ± 17.6		41.9 ± 20.2	
*Interviewed*			**0.02**		0.9		**< 0.0005**
No (anonymous)	199(79.9)	47.2 ± 18.2		25.8 ± 19.6		28.3 ± 21.5	
Yes	50 (20.1)	53.9 ± 15.8		25.5 ± 19.7		45.5 ± 22.7	

The mean knowledge score of all participants was 48.5 ± 17.9. Knowledge score was significantly higher among physicians who prescribe cannabis than it was among those who do not (*p* = 0.002), among those who encounter patients with SUD daily (*p* < 0.0005), and among physicians younger than age 65 (49.4 ± 17.7 compared with 43.1 ± 18.1, *p* = 0.05). The score was also higher among physicians who were interviewed than it was among the anonymous respondents (*p* = 0.02). Higher score characterized physicians who self-reported partial knowledge about OMT (*p* < 0.0005) and ability to identify individuals with SUD (*p* < 0.0005). Comparison of participants by specialty revealed the highest score among pain medicine physicians (57.2 ± 14), followed by psychiatrists (56.8 ± 16.8), family medicine physicians (51.5 ± 16.3), internal medicine physicians (46.9 ± 14.6), neurologists (46.3 ± 16), and surgeons (39.9 ± 18.6). This was not related to gender, place of study medicine, managerial position, or workplace (community or hospital). The knowledge score did not differ between prescribed to non-prescribed opioids. For example, 66.9% of opioid prescribers vs. 54% of non-prescribers correctly agreed that prescription for a limited period reduces the risk of OUD. On the other hand, only 65.1% of opioid prescribers vs. 74.2% of non-prescribers correctly agreed that opioids taken “as needed” increase the risk of OUD.

#### *Approach score (*Table 4*)*

The mean approach score of all participants was 31.7 ± 2.8. The score was higher among those in the 45–64 age group (*p* = 0.05), among physicians with a managerial role (*p* = 0.05), among physicians who prescribe opioids (*p* < 0.0005), and among physicians who prescribe cannabis (*p* < 0.0005). A higher approach score was also found among physicians who work in the community (only or in addition to hospital work) (34.3 ± 24) than among physicians who work only in a hospital (28.5 ± 20.8, *p* = 0.05). Higher score characterized physicians who self-reported knowledge about OMT (*p* < 0.0005) and knowledge to identify individuals with SUD (*p* < 0.0005). Out of the various specialties, the highest mean approach scores were found among pain physicians (50.7 ± 20.8) and family medicine physicians (49.8 ± 21.8). The approach score was higher among physicians who were interviewed, than it was among those who responded to the anonymous questionnaire (*p* < 0.0005).

#### *Stigma score (*Table 4*)*

The mean stigma score of all participants was 25.7 ± 19.6. The score was higher among physicians who prescribe opioids (*p* = 0.03). For instance, 42.5% of them compared with 34.8% of the non-prescribers, thought that OMT means replacing one addiction with another. Higher score characterized physicians who self-reported partial knowledge about OMT (*p* < 0.0005) and knowledge to identify individuals with SUD (*p* < 0.0005). Although there was no significant difference in stigma score among physicians from the various specialties, the highest stigma score was found among pain physicians (34.1 ± 22.6), and among a small group of “other physicians” (family and internal medicine).

#### Multivariate analyses

**Fig. 1:**
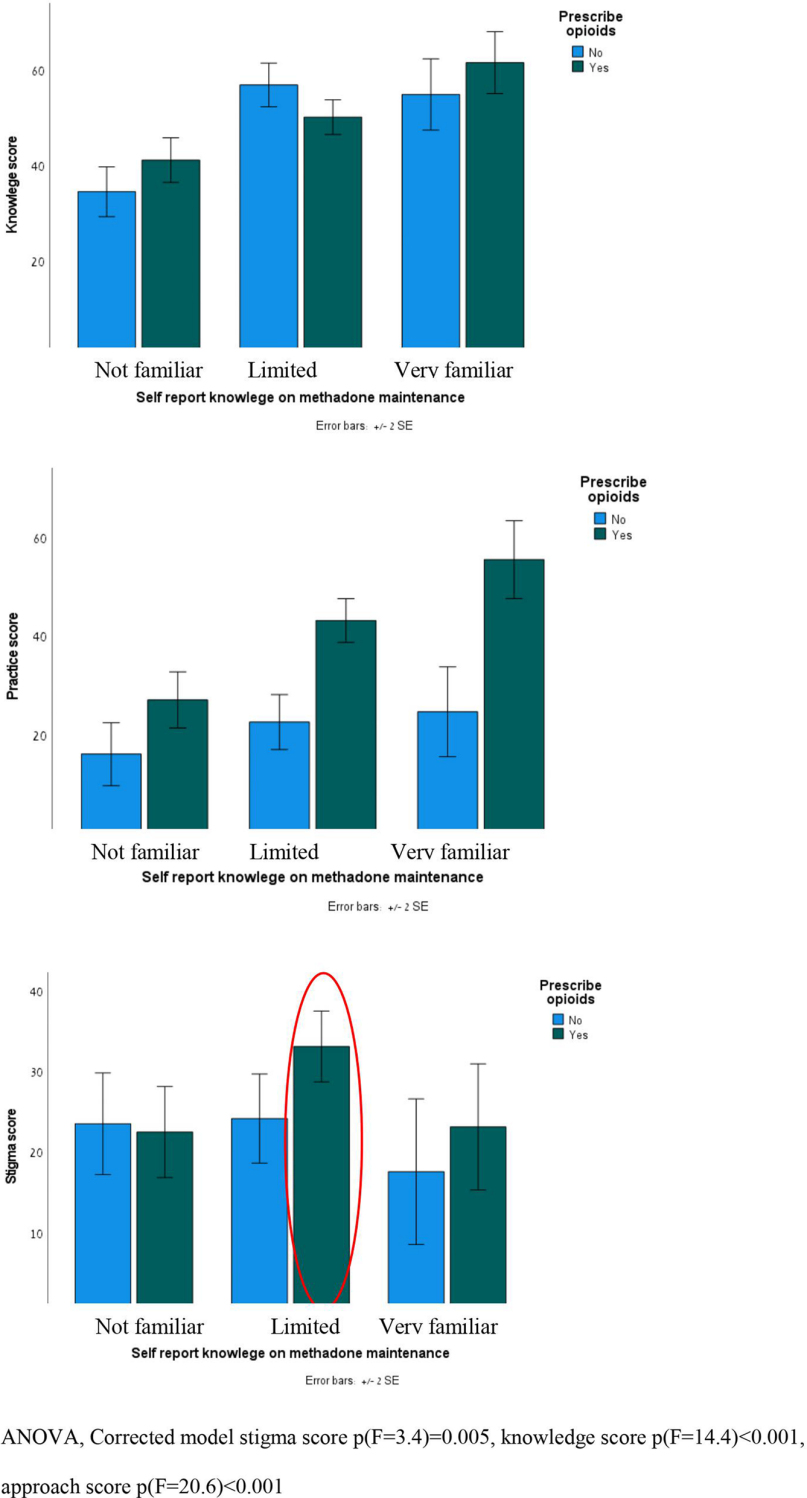
Knowledge, approach, and stigma scores, by “prescribe opioids” and self-reported knowledge about methadone maintenance treatment. ANOVA, corrected model stigma score *p*(*F* = 3.4) = 0.005, knowledge score *p*(*F* = 14.4) < 0.001, and approach score *p*(*F* = 20.6) < 0.001

Multivariate analyses with knowledge, approach, and stigma scores as dependent variables, including independent variables with *p* < 0.1 (Table 4) found significant the two variables “self-reported knowledge about OMT” and “prescribing opioids” (Fig. 1). The results imply that knowledge and approach scores are higher among those with greater knowledge about OMT, and among those who prescribe opioids. The highest stigma scores were found among the 76 physicians who prescribe opioids but report having limited knowledge regarding OMT (Fig. 1, highlighted in red). ANOVA, corrected model stigma score *p*(*F* = 3.4) = 0.005, knowledge score *p*(*F* = 14.4) < 0.001, approach score *p*(*F* = 20.6) < 0.001.

#### Logistic regression

The 75th percentile knowledge score was 60 (75% of the participants scored below 60 “low score,” and 25% scored 60 or above “high score”), the 75th percentile approach score was 40 and the 75th percentile stigma score was 35. Logistical regression models that included all those variables that were found to be with *p* < 0.1 in univariate analyses (Table 5) found that (Table 6) having a high knowledge score (scored ≥ 60) were those who encountered patients with SUD on a daily basis (OR = 3.5, 95% CI 1.7–7.1), those who were very familiar with OMT (OR = 10.1, 95% CI 3.5–29.0), and those who had limited familiarity with OMT (OR = 4.6, 95% CI 1.8–11.7). Having a high stigma score (≥ 35) were physicians who prescribed opioids (OR = 1.7, 95% CI 1.0–2.9) and physicians who reported having limited knowledge about OMT (OR = 2, 95% CI 1.1–3.7). Physicians with a high approach score (≥ 40) were those who prescribe opioids (OR = 11.7, 95% CI 4.9–28), prescribe cannabis (OR = 2.1, 95% CI 1.0–4.3), are very familiar with identifying SUD (OR = 32.5, 95% CI 4.1–260), have limited knowledge in identifying SUD (OR = 11.2, 95% CI 1.4–89), and who were interviewed and did not respond to the anonymous questionnaire (OR = 3.6, 95% CI 1.6–7.9).

**Table 5:** Knowledge, stigma, and approach scores in the 75th percentile, by selected variables

	*N* (%)	Knowledge score ≥ 60	*p* value	Stigma score ≥ 35	*p* value	Approach score ≥ 40	*p* value
All sample	249 (100%)	63 (25.3%)		91 (36.5%)		75 (30.1%)	
*Medicine studied*			0.07		0.6		0.6
Israel	182 (73.1)	52 (28.6)		69 (37.9)		53 (29.1)	
Others	67 (26.9)	11 (16.4)		22 (32.8)		22 (32.8)	
*Gender*			0.5		0.4		0.3
Female	112 (45.0)	31 (27.7)		44 (39.3)		30 (26.8)	
Male	137 (55.0)	32 (23.4)		47 (34.3)		45 (32.8)	
*Age group*			0.5		0.7		0.2
<45	86 (34.5)	21 (24.4)		29 (33.7)		25 (29.1)	
45-64	122 (49.0)	34 (27.9)		45 (36.9)		42 (34.4)	
65+	41 (16.5)	8 (19.5)		17 (41.5)		8 (19.5)	
*Manager*			0.2		0.3		0.1
Yes	127 (51)	37 (29.1)		42 (33.1)		44 (34.6)	
No	122 (49)	26 (21.3)		49 (40.2)		31 (25.4)	
*Meet daily SUD*			**<0.0005**		0.2		0.3
Yes	46 (18.5)	24 (52.2)		21 (45.7)		17 (37.0)	
No	203 (81.5)	39 (19.2)		70 (34.5)		58 (28.6)	
*Prescribe opioids*			0.5		0.05		**< 0.0005**
Yes	146 (58.6)	34 (23.3)		61 (41.8)		67 (45.9)	
No	103 (41.4)	29 (28.2)		30 (29.1)		8 (7.8)	
*Prescribe cannabis *			0.2		0.7		**< 0.0005**
Yes	82 (32.9)	25 (30.5)		28 (34.1)		35 (42.7)	
No	167 (67.1)	38 (22.8)		63 (37.7)		40 (24.0)	
*Specialty*			**<0.0005**		0.07		**<0.0005**
Psychiatry	62	28 (45.2)		17 (27.4)		6 (9.7)	
Surgery	31	4 (12.9)		11 (35.5)		2 (6.5)	
Internal medicine	48	8 (16.7)		23 (47.9)		18 (37.5)	
Pain medicine	22	9 (40.9)		11 (50)		15 (68.2)	
Family medicine	34	11 (32.4)		16 (47.1)		22 (64.7)	
Oncology	6	0		0		1 (16.7)	
Geriatric/rehab/*	25	2 (8)		7 (28)		7 (28)	
Neurology	10	1 (10)		2 (20)		0	
Other	3	0		1 (33)		2 (66.7)	
Pediatrics	8	0		3 (37.5)		2 (25.0)	
*Working place*			0.5		1.0		0.1
Community clinic	70 )28.1)	20 (28.6)		26 (37.1)		26 (37.1)	
Hospital	110 (44.2)	24 (21.8)		40 (36.4)		26 (23.6)	
Both	69 (27.7)	19 (27.5)		25 (36.2)		23 (33.3)	
*Self-report: identify*			**<0.0005**		0.3		**< 0.0005**
No	39 (15.7)	1 (2.6)		11 (28.2)		1 (2.6)	
Partial	106 (42.6)	21 (19.8)		37 (34.9)		26 (24.5)	
Yes	104 (41.8)	41 (39.4)		43 (41.3)		48 (46.2)	
*Self-report: OMT *			**<0.0005**		**0.007**		**< 0.0005**
No	83)33.3)	6 (7.2)		24 (28.9)		12 (14.5)	
Partial	124 (49.8)	36 (29)		57 (46.0)		41 (33.1)	
Yes	42 (16.9)	21 (50)		10 (23.8)		22 (52.4)	
*Interviewed*			0.1		1.0		**< 0.0005**
No (anonymous)	199 (79.9)	46 (23.1)		73 (36.7)		45 (22.6)	
Yes	50 (20.1)	17 (34)		18 (36)		30 (60.0)

**Table 6:** Logistic regression models for knowledge, stigma, and approach

	Odds ratio (95% CI)	*P* value
*Knowledge score 60* +		
Meet daily SUD	3.4 (1.7–7.1)	**< 0.001**
Self-report OMT-Familiar	10.1 (3.5–29.0	**< 0.001**
Self-report OMT-Partial	4.6 (1.8–11.7)	**0.001**
*Stigma score 35* +		
Prescribe opioids	1.7 (1.0–2.9)	0.05
Self-report OMT-Partial	2.0 (1.1–3.7)	**0.018**
*Approach score 40* +		
Prescribe opioids	11.7 (4.9–28.0)	**< 0.001**
Prescribe cannabis	2.1 (1.0–4.3)	0.05
Self-report OMT-Partial	11.2 (1.4–89.0)	**0.02**
Self-report: Identify SUD	32.5 (4.1–260)	**0.001**
Interviewed (not anonymous)	3.6 (1.6–7.9)	**0.001**

## Discussion

We conducted the current survey to evaluate physicians’ objective knowledge, level of stigma and approach regarding the prescribing of opioids, self-reported familiarity with SUD risk factors, identification of patients with SUD, and knowledge about OMT for OUD. The knowledge score showed no difference between those who prescribe opioids and those who do not in our survey. A study from Taiwan studying prescribing opioids for chronic noncancerous pain found that non-pain physicians had a significantly lower knowledge level, more negative attitudes, and greater hesitation about prescribing opioids compared to the pain-related physicians [28]. Thus, our sample is heterogeneous, including different specialties that differed in their knowledge scores. The physicians’ specialties differed in their proportions of prescribing, and their indications (i.e., acute pain is most likely prescribed by surgeons or orthopedics). When we compared knowledge scores by prescription indication (data not shown), we found no differences, which may be due to the small sample size, but also may relate to the fact that scores covered several aspects that differed between the specialties.

Opioid prescribing was found to be associated with a high-grade approach score, was not found to be related to knowledge, but was related to stigma. Similarly, a previous survey found that negative attitudes toward patients who misuse opioids were common among physicians, as were personal experiences of bias toward this patient population [25].

The hypothesis that lack of knowledge would be related to stigma, specifically stigma and erroneous beliefs, was not broadly demonstrated here. Among pain medicine specialists, the knowledge and stigma scores were relatively high, compared to those of other physicians. We previously found an inverse relationship between knowledge and stigma toward OMT among healthcare workers in the field of addiction medicine [20].

Like our past findings, physicians in the present study who reported encountering patients who suffer from SUD daily, had a particularly high knowledge score, but had no difference in their stigma score, which we would have expected to be lower. Half of the physicians who reported encountering patients who suffer from SUD daily were psychiatrists whose stigma score was lower. However, because the rest of these physicians were primarily internal medicine specialists and surgeons, the mean stigma score was not low. An online questionnaire survey from Nova Scotia that also studied all the three aspects, contrary to our results, found poor knowledge to be associated with stigma [29]. Specifically, they showed that 43% of family physicians in the community were “unwilling” to prescribe methadone for people with SUD, mainly because of lack of knowledge about OMT, lack of experience with SUD, and their perception that these were “difficult” patients. The belief that addiction is a response to psychological woundedness, or a result of moral failings, was prevalent among primary care physicians and psychiatrists [30]. Knowledge and approach however were not evaluated in that study.

The impact of access to addiction specialists on attitudes, beliefs, and hospital-based clinical practices regarding OUD was studied in a survey of hospitalist physicians in the USA [32]. While half of 262 responders rarely or never screened for OUD, and fewer than 10% initiated buprenorphine, hospitalists with access to addiction specialists were more likely to feel supported to screen and refer patients to treatment [32]. The results of our study are consistent and may reflect the reports [22, 23, 33] that over 60% of patients suffering from SUD who are receiving OMT suffer from stigma, including receiving unfair treatment and the feeling that physicians are afraid of them. The findings of our survey led to the characterization and identification of physicians who need guidance in the prevention and treatment of OUD. Some physicians require only knowledge guidance, while others require assistance and guidance to influence their negative stigma and beliefs about OMT. Our findings are no different than those of published studies conducted in other parts of the world. For example, a similar survey conducted among dentists in the USA showed similar findings [34], and recommended including the topic in the dental and medical curricula.

Data on the growing number of opioid users and opioid-related deaths are a major cause for concern. Specifically, the data on the increase in hospitalizations in a public setting for acute intoxication due to substance use, relative to previous years, are alarming. In Israel, the most common substance of abuse is opioids (both prescription medications and heroin) (*n* = 1097), followed by alcohol (*n* = 970) and cannabis (*n* = 947) [9]. At the same time, there was a dramatic increase in the licenses for cannabis for medical use between 2019 and 2022 (from 30,000 to above 100,000), with more than a third of these prescriptions being for non-cancer chronic pain [35]. In addition, negative beliefs and a lack of knowledge and experience regarding OMT are well-documented in the medical literature and are responsible, at least in part, for the low rate of physician referrals to this treatment, and of patients who seek this treatment.

Based on the findings in this study, it may be concluded that there is a need for a comprehensive instructional program to increase knowledge both about pain medication, and about SUD. Learning to recognize signs of substance use disorder, knowledge about OMT that would reduce stigma, and knowing where to refer patients in need of treatment are all crucial to the treatment of pain, and are equally if not more important to family medicine and internal medicine physicians, who frequently encounter patients at risk of SUD.

By imparting knowledge about OMT, which is the most effective treatment for OUD [36], physicians who encounter patients with OUD can refer them for appropriate treatment and expedite their entrance into treatment, thereby increasing their safety and preventing further harm. Education regarding the referral of patients to OMT programs should be emphasized and encouraged, particularly among pain medicine physicians, who often do not refer patients due to stigma. In the case of family medicine and internal medicine physicians, many of whom encounter patients with SUD, stigma can interfere with the physician–patient relationship.

## Study limitations

Despite the representativeness of our sample to the entire physician population in Israel [37], we treat with seriousness the low response rate, and that the respondents represent selected physicians who are interested in this important issue. If other physicians who are less interested or even less knowledgeable had participated in this study, then the results might have shown even lower scores. Such a low response rate of physicians has been reported elsewhere, even though, unlike in our survey, those physicians were offered a reward for participating [25].

Another limitation is the 50 physicians in managerial positions who work in relevant specialties in hospitals and in the community and whom we interviewed. Their scores for knowledge and approach were high, most likely since only managers were selected for interviews. However, their level of stigma was comparable to that of the others, and not low, as we had expected. We studied them to enhance the validity of the anonymous responses, which may differ from the personal responses. Moreover, as our hypothesis was of poor knowledge and high stigma, we interviewed physicians in managerial positions to err on the side of caution.

Hence, our findings most likely describe “the tip of the iceberg” of the physician population and generate an unequivocal recommendation to increase knowledge that will reduce stigma and save lives. We must limit our findings to the development and use of a new tool, rather than a previously validated one. Thus, future studies are needed to validate this tool, as well.

### Supplementary Information

**Additional file 1**. Appendix.

## Data Availability

The datasets used and/or analyzed during the current study are available from the corresponding author upon reasonable request.
