# Supplementary material for: Stigma and level of familiarity with opioid maintenance treatment (OMT) among specialist physicians in Israel
Source: Harm Reduct J. 2023 Sep 15;20:134. doi: 10.1186/s12954-023-00869-9 (PMC10503015; doi:10.1186/s12954-023-00869-9)
Supplement: Supplementary file 1 — Additional file 1. Appendix. [file 12954_2023_869_MOESM1_ESM.docx]

**Questionnaire**

**Greetings,**

**In light of the opioid epidemic in the USA, and in order to try to prevent a similar situation from developing in Israel, we are interested in learning about the attitudes and prescribing behaviors of doctors in Israel regarding opioids and cannabis in order to build a training program to help educate physicians regarding best practices.**

**Thank you for your cooperation in filling out an anonymous questionnaire.**

The details are intended for research purposes only; the questionnaire is worded in the masculine but is intended for all genders.

Thank you in advance for your cooperation!

1. **Position:** attending physician/ resident/ intern.
2. **Place of study:** Israel, Eastern Europe, Western Europe, USA or Canada, other:_______.
3. **Specialization:** pain medicine/ oncology/ hematology/ family medicine/ emergency room/ surgery/ internal medicine/ rheumatology/ orthopedics/ obstetrics and gynecology / pediatrics/ ENT/ dermatology (skin and sexually transmitted diseases)/ ophthalmology/ gastroenterology/ psychiatry/ neurology/ rehabilitation/ urology/ other : ____________.
4. **Place of work:** 1. hospital, 2. community. Detail: __________.
5. **Seniority in the medical field (years):** __________.
6. **Management position**: yes/no.
7. **Full-time/part-time position:** _____________.
8. **Age:** __________.
9. **Gender:** Male/Female/Other.
10. **Country of birth:** Israel, Eastern Europe, Western Europe, North America, South America, Australia/New Zealand, other:_______________.
11. **Year of immigration:** _________.
12. **How many opioid prescriptions do you write in a month?**A. 0, b. 1-5, c. 5-10, d. 10-20, e. 20-30 and Other _____________.
13. **Opioids are mainly prescribed for:**
    A. Acute pain, b. Chronic pain that is not of cancerous origin, c. Patients with malignancies. d. Other ___________.
14. **What is the frequency with which you give prescriptions/recommendations for medical cannabis during a month?**A. 0, b. 1-5, c. 5-10, d. 10-20 e. 20-30 and Other _____________**.**
15. **I recommend medical cannabis mainly for:**A. Chronic pain that is not of cancerous origin b. Neurological diseases c. Psychiatric disorders d. Cancer/HIV patients**.**
16. **How would you define addiction (check the correct answer):**A. Mental disorder
    B. Behavioral disease
    C. A disease with genetic and biological components
    D. All of the answers are correct
17. **Do you encounter patients who suffer from addiction in your work?**A. Not at all, b. On a daily basis, c. Several times a month, d. Several times a year e. Other _____________.
18. **Do you encounter patients who suffer from opioid use disorder in your work?**A. Not at all, b. On a daily basis, c. Several times a month, d. Several times a year e. Other _____________.
19. **If you encounter a patient who suffers from opioid use disorder, do you take any steps? (check all that apply)**

| A. Does not happen/ not relevant |  |
| --- | --- |
| B. It's not one of my therapeutic areas, I don't do anything |  |
| C. I don't have knowledge in this field, I don't do anything |  |
| D. Suggest the patient contact the doctor who gave him opioids for further management | approach |
| E. Suggest the patient seek out addiction treatment | approach |
| F. Explain the disease of addiction to the patient | approach |
| G. Increase the frequency of monitoring the patient | approach |
| F. Help him to stop using opioids myself | approach |
| H. Offer him a hepatitis B vaccine | approach |
| I. Offer him a hepatitis C test, and if necessary, treatment | approach |

1. **If your patient developed addiction to opioids as a result of the medication you prescribed, what would you do? (check all that apply)**

| A. Does not happen/ not relevant |  |
| --- | --- |
| B. Reduce the dose and intensive monitoring | approach |
| C. Continue to give him the medication unchanged |  |
| D. Suggest the patient seek addiction treatment | approach |
| E. Other: ____________ | approach |

1. **Do you find out / check / consider - risk of addiction before giving opioids to patients?**

| A. Does not happen/ not relevant |  |
| --- | --- |
| B. Yes | approach |
| C. No, because there is no alternative |  |
| D. No, because it is impossible to predict |  |
| E. No, because I have no knowledge about addiction |  |
| F. Other: ____________ |  |

1. **After prescribing opioids, do you conduct follow-up on the patient, and if so how?**

| A. Does not happen/ not relevant |  |
| --- | --- |
| B. Do not conduct follow-up |  |
| C. Do not follow, but refer to a family doctor/other |  |
| D. After stabilization on a fixed dose, follow-up every 3-6 months | approach |
| E. After stabilization on a fixed dose, follow-up every 12 months |  |
| F. Other: ____________ |  |

1. **Who manages your patients' opioid prescriptions?**

| A. Does not happen/ not relevant |  |
| --- | --- |
| B. One doctor who manages all of their medications | approach |
| C. A pain specialist | approach |
| D. Not managed |  |
| E. Do not know |  |
| F. Other: ____________ |  |

1. **Where would you refer a patient who suffers from opioid use disorder or who has developed an addiction to opioids due to treatment: (Rate all the options between 1-5 where 1=do not agree at all and 5=agree to a large extent):**

|  | 1 | 2 | 3 | 4 | 5 |
| --- | --- | --- | --- | --- | --- |
| A. To a detoxification center |  |  |  |  |  |
| B. To an opioid treatment program for buprenorphine maintenance treatment |  |  |  |  |  |
| C. To an opioid treatment program for methadone maintenance treatment |  |  |  |  |  |
| D. To a psychiatrist |  |  |  |  |  |
| E. To a pain clinic |  |  |  |  |  |
| F. To the emergency room |  |  |  |  |  |
| G. To a psychologist |  |  |  |  |  |
| H. To a social worker |  |  |  |  |  |
| I. To a treatment center for street drug users (rehab) |  |  |  |  |  |
| J. To an alternative treatment |  |  |  |  |  |
| K. Other: ____________ |  |  |  |  |  |

1. **Rate your level of knowledge on the following subjects (1 = not familiar, and 5 = very familiar):**

|  | 1 | 2 | 3 | 4 | 5 |
| --- | --- | --- | --- | --- | --- |
| A. Opioid treatment for chronic non-cancer pain |  |  |  |  |  |
| B. Identification and diagnosis of addiction disorder |  |  |  |  |  |
| C. Opioid addiction treatment options |  |  |  |  |  |
| D. Methadone maintenance treatment |  |  |  |  |  |
| E. Maintenance treatment with buprenorphine (Subutex/Suboxone) |  |  |  |  |  |

1. **Indicate for each of the following statements the degree of your agreement (choose 1-5 where 1= do not agree at all and 5= agree to a large extent, indicate 0 if not applicable/don't know):**

| Q |  | 0 | 1-5 |  |
| --- | --- | --- | --- | --- |
| 1 | Methadone maintenance treatment is only a step on the way to full recovery |  | 1-5 | stigma |
| 2 | A person with an addiction disorder can be recognized externally |  | 1-5 | stigma |
| 3 | Cannabis use may lead to an outbreak of psychotic symptoms |  | 1-5 | knowledge |
| 4 | A patient who requests increasing amounts of medicine due to ineffectiveness in treating pain raises the suspicion of developing an opioid use disorder |  | **5-1** | knowledge **opposite** |
| 5 | Methadone maintenance treatment means replacing one addiction with another |  | 1-5 | stigma |
| 6 | A patient with a history of depression and anxiety is at risk of developing addiction |  | 1-5 | knowledge |
| 7 | When I encounter a person who suffers from opioid use disorder, I feel disgust and aversion towards that person |  | 1-5 | stigma |
| 8 | When I prescribe cannabis treatment, I consider the risk of developing an addiction |  | 1-5 | knowledge |
| 9 | Methadone maintenance treatment reduces the risk of infection with infectious diseases (HIV and HCV) due to the reduction of injections |  | 1-5 | knowledge |
| 10 | A patient who repeatedly requests an increase in the dose while his functioning continues to deteriorate raises the suspicion of suffering from opioid use disorder |  | 1-5 | knowledge |
| 11 | Taking an opioid for a limited period reduces the chance of developing opioid use disorder |  | 1-5 | knowledge |
| 12 | Retention in methadone maintenance treatment after one year is higher than 70% |  | 1-5 | knowledge |
| 13 | A patient who describes symptoms of physical withdrawal after a break in pain treatment raises the suspicion of suffering from opioid use disorder |  | **5-1** | knowledge **opposite** |
| 14 | Methadone maintenance treatment causes euphoria |  | **5-1** | knowledge **opposite** |
| 15 | Before giving opioids, the risk of developing and/or aggravating mental disorders must be considered |  | 1-5 | knowledge |
| 16 | Taking an opioid as needed increases the risk of developing addiction in patients at risk |  | 1-5 | knowledge |
| 17 | A patient who frequently requests prescriptions because he has lost them or who turns to other doctors for various reasons to request the same prescriptions, raises the suspicion of suffering from opioid use disorder |  | 1-5 | knowledge |
| 18 | After detoxification (without maintenance treatment), patients with addiction to opioids have an 80% chance of returning to use |  | 1-5 | knowledge |
| 19 | I treat patients who suffer from substance use disorders differently |  | 1-5 | stigma |
| 20 | Methadone maintenance treatment can only be started after failed attempts at detoxification in a rehabilitation center |  | 1-5 | knowledge |
| 21 | Addiction is a chronic disease like other chronic diseases |  | **5-1** | stigma  **opposite** |
| 22 | A pregnant woman using cannabis may cause cognitive damage to the fetus |  | 1-5 | knowledge |
| 23 | Taking an opioid in preparations with a long half-life increases the risk of developing addiction |  | **5-1** | knowledge  **opposite** |
| 24 | When I encounter a person who suffers from substance use disorder, I feel helpless and despair about the likelihood of the success of the treatment |  | 1-5 | stigma |
| 25 | The patients in maintenance treatment with methadone are more "problematic" and violent than patients who go through rehabilitation without maintenance treatment |  | 1-5 | stigma |
| 26 | Using marijuana for pain may improve depression and anxiety disorders |  | **5-1** | knowledge **opposite** |
| 27 | The recommended maintenance treatment with methadone is chronic treatment for many years |  | 1-5 | knowledge |
| 28 | Medical cannabis given as pain treatment is not addictive |  | **5-1** | knowledge **opposite** |
| 29 | A patient with a history of neglect and sexual abuse in childhood is at increased risk of developing addiction |  | 1-5 | knowledge |
| 30 | Patients in methadone maintenance treatment do not need painkillers |  | **5-1** | knowledge **opposite** |
| 31 | Methadone maintenance treatment reduces cravings for opioids |  | 1-5 | knowledge |
| 32 | Before prescribing cannabis, I consider the risk of developing and/or aggravating mental disorders |  | 1-5 | knowledge |
| 33 | Methadone maintenance normalizes the HPA (Hypothalamus Pituitary Adrenal) axis |  | 1-5 | knowledge |
| 34 | A patient who suffers from ADHD and reports medication treatment with Ritalin in childhood is at increased risk of developing addiction |  | **5-1** | Knowledge **opposite** |
| 35 | Taking an opioid at regular times may reduce the risk of developing addiction |  | 1-5 | knowledge |
| 36 | Methadone maintenance treatment prevents opioid withdrawal syndrome |  | 1-5 | knowledge |
| 37 | A patient who has a family history of addiction is at increased risk of developing addiction |  | 1-5 | knowledge |
| 38 | Cocaine addiction, without concurrent opioid addiction, can be treated with methadone maintenance treatment |  | 1-5 | knowledge |
| 39 | Methadone maintenance treatment is not intended for pregnant women who suffer from opioid use disorder |  | 1-5 | knowledge |

**Thank you very much for your cooperation!**
